# Do Postoperative Telehealth Visits Require a High Rate of Redundant In-Person Evaluation After Upper Extremity Surgery?

**DOI:** 10.7759/cureus.21462

**Published:** 2022-01-20

**Authors:** Tyler W Henry, Arlene Maheu, Samir Sodha, Moody Kwok, Greg G Gallant, Pedro Beredjiklian

**Affiliations:** 1 Orthopaedic Surgery, Rothman Orthopaedic Institute, Philadelphia, USA; 2 Orthopaedic Surgery, Rothman Orthopaedic Institute, New Jersey, USA; 3 Orthopaedic Surgery, Hackensack University Medical Center, New York, USA; 4 Division of Hand Surgery, Rothman Orthopaedic Institute, Philadelphia, USA

**Keywords:** complications, visits, postoperative, upper extremity, telehealth

## Abstract

Background

Telehealth platforms may save resources for patients and providers, but the precise impact of their incorporation during the postoperative period is not well understood. The goal of this study is to determine whether telehealth incorporation in the postoperative period leads to an overall increase in healthcare utilization after upper extremity surgery.

Methodology

Patients seen for a postoperative telehealth visit after upper extremity surgery were randomly selected and retrospectively enrolled. Complications and the total number of postoperative visits before clinical discharge were recorded and compared to controls matched by surgery type and surgeon.

Results

A total of 56 patients were seen for 60 telehealth visits. The most common surgical procedures were distal radius open-reduction internal fixation (n = 8), open carpal tunnel release (n = 8), and endoscopic carpal tunnel release (n = 6). One telehealth visit (1.7%) required conversion to in-person evaluation due to suspected superficial infection necessitating in-person physical examination. The average number of postoperative visits prior to clinical discharge was 2.6 in the telehealth group compared to 2.7 in matched controls (p = 0.886). Complication rates were similar between groups.

Conclusions

The rate of necessary in-person evaluation after postoperative telehealth visits was less than 2%. The incorporation of telehealth visits did not appear to increase healthcare utilization after upper extremity surgery. Accordingly, the postoperative period is likely an ideal application for safe and effective telehealth implementation.

## Introduction

As telehealth incorporation continues to rapidly expand across orthopedic practices [1,2], it remains unclear which clinical scenarios are the most and least amenable to virtual visits [3]. Among them, postoperative telehealth visits have proven to be safe [4], but there is a paucity of data in the hand surgery literature. Moreover, their impact on the broader postoperative course is incompletely understood. If a substantial percentage of encountered postoperative visits cannot be managed virtually, telehealth visits would carry a high rate of necessary conversion to in-person evaluation, thus creating redundant clinical visits often within a global billing period. This would, on average, increase the number of visits performed within the postoperative course, effectively costing rather than saving resources.

If the inherent limitations of telehealthcare delivery trigger a high rate of necessary conversion to in-person evaluation, its utility in saving resources during the postoperative period would be unclear. The goal of this study is to assess postoperative healthcare utilization in patients seen via telehealth for at least one clinical visit after upper extremity surgery and determine the conversion rate to in-person evaluation. We hypothesized the in-person conversion rate to be minimal, and that postoperative healthcare utilization would be similar to matched controls.

## Materials and methods

After Thomas Jefferson University Institutional Review Board approval (#13D.432), with a waiver of informed consent per institutional protocol, a database query was performed to identify all patients seen for a postoperative telehealth visit (CPT code: 99024) between April and May 2020 within the hand and upper extremity division of a single, large orthopedic practice. Electronic medical records were reviewed, and patients lacking follow-up through clinical discharge (defined as the clinical visit at which the treating surgeon released the patient to as-needed follow-up) were excluded. Patient demographics were recorded in addition to the surgical procedure code, the total number of postoperative visits, days to clinical discharge, complications, and reoperations. Details of the individual telehealth visits were also tracked and recorded, including the modality used (audiovisual versus audio only), any specific changes to the postoperative treatment course made during the visit, frequency of radiographic evaluation incorporation, and any therapeutic interventions administered during the visit.

The rate of telehealth visits requiring conversion to an in-person evaluation and the mean number of visits within each patient’s postoperative course compared to matched in-person controls were primarily assessed. A conversion to in-person evaluation was considered to be any telehealth visit during which the patient was instructed to schedule an in-person evaluation to further address a specific complaint or concern due to the inherent limitations of virtual encounters (i.e., inadequate physical examination). Complication and reoperation rates were secondarily compared between the telehealth and control groups.

A power analysis was performed to identify an effect size of 0.55 at a power of 0.80 and 95% significance level with nonparametric mean testing of the total number of postoperative visits between the telehealth and control groups. Accordingly, 56 patients seen for a postoperative telehealth visit were selected using a random number generating sequence and matched 1:1 to a group of controls based on surgeon and surgical procedure code selected using a random number generating sequence. All matched control surgical procedures occurred between 2018 and 2020. Electronic medical records were reviewed to assess the postoperative course of matched controls and confirm that the included patients were seen in person for all postoperative visits.

All data were recorded and analyzed using the Statistical Package for the Social Sciences (SPSS Inc, Ver 26.0; IBM Corp., Armonk, NY, USA). The Mann-Whitney U test was used for testing of mean differences, and the chi-square test was used to compare the rates of complications and reoperations between the telehealth and control groups. Statistical significance was maintained at p-values of <0.05.

## Results

**Figure 1:**
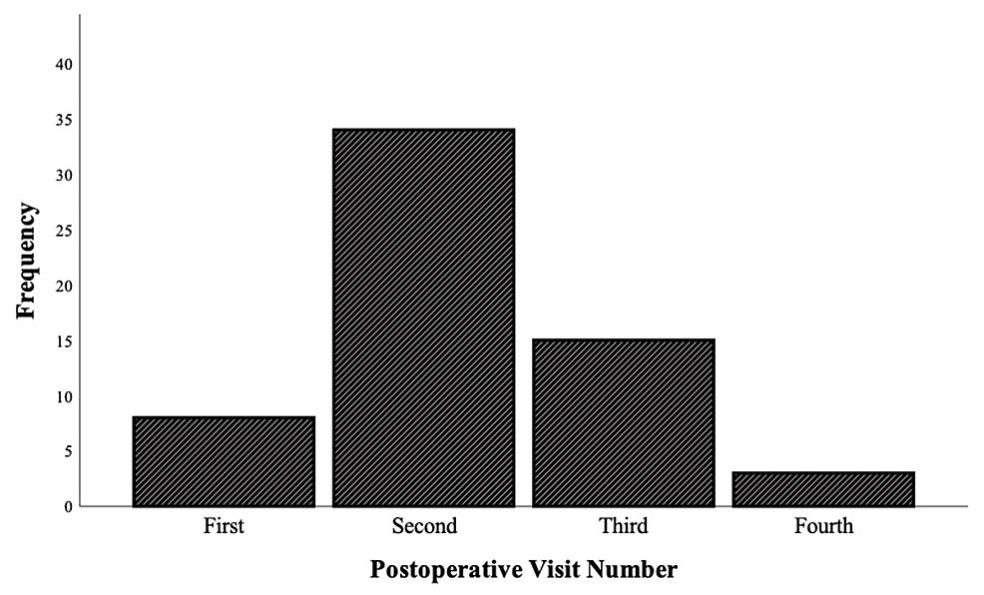
Postoperative visit number frequency for the 60 telehealth visits conducted within the study population.

A total of 56 patients were seen for 60 telehealth visits during the study period. The mean time from surgery to telehealth visit was 60.4 days (range: 5-129 days; standard deviation (SD) = 28.7), and the telehealth encounter was most commonly the second postoperative visit (Figure 1). There were 32 women and 24 men with an average age of 59 years (range: 21-84 years; SD = 14.8). The most common surgical procedures were distal radius open-reduction internal fixation (CPT code: 25607/25609) (n = 8), open carpal tunnel release (CPT code: 64721) (n = 8), and endoscopic carpal tunnel release (CPT code: 29848) (n = 6) (Table 1). All surgeries were performed, and telehealth visits were conducted by one of 12 fellowship-trained upper extremity surgeons. In total, 55 (91.7%) visits were conducted through audiovisual platforms and five (8.3%) visits were audio-only. Radiographs were obtained prior and reviewed during the encounter in nine (15.0%) visits. Suture removal instructions were given, and patient removal was performed, in four (6.7%) initial postoperative visits. A specific change to the postoperative treatment course solely based on the findings of the telehealth visit was made in three (5.0%) instances. These included oral antibiotic therapy initiation, physical therapy initiation, and oral steroid initiation. Patients were directly discharged to as-needed follow-up from 37 (61.7%) telehealth visits, and the remaining 23 (38.3%) visits were followed by a subsequent telehealth or in-person visit.

**Table 1:** Index surgical procedure frequency for the 56 patients seen for at least one telehealth visit during the study period. Note: Procedure frequencies also reflect the 56 controls, equally matched by CPT code and surgeon.

CPT code	Procedure description	Frequency
64721	Open carpal tunnel release	8
25607/25609	Distal radius open-reduction internal fixation	8
29848	Endoscopic carpal tunnel release	6
26055	Trigger finger release	5
25447	Carpometacarpal joint arthroplasty	5
25000	DeQuervain’s release	3
26432	Mallet finger percutaneous pinning	2
23615	Proximal humerus open-reduction internal fixation	1
24342	Distal biceps tendon repair	1
24359	Lateral epicondyle debridement and repair	1
25210	Hook of hamate excision	1
25825	Wrist arthrodesis	1
26055, 26121	Trigger finger release and Dupuytren’s contracture release	1
26116	Soft tissue mass excision	1
26145	Tenosynovectomy	1
26160	Mucous cyst excision	1
26230	Exostectomy	1
26531	Metacarpophalangeal joint arthrodesis	1
26540	Ulnar collateral ligament repair	1
26727	Phalanx closed-reduction percutaneous pinning	1
26735	Phalanx open-reduction internal fixation	1
29827	Arthroscopic rotator cuff repair	1
29846	Triangular fibrocartilage complex repair	1
64718	Cubital tunnel decompression	1
64718, 64721	Cubital tunnel decompression and carpal tunnel release	1
64721, 26055	Carpal tunnel release and trigger finger release	1

One (1.7%) telehealth visit required conversion to in-person evaluation due to suspected superficial infection necessitating an in-depth physical examination. The patient was started on oral antibiotics at the time of the telehealth visit, and the infection ultimately cleared without surgical intervention.

There were no significant differences in the mean age between the telehealth cohort (59 years) and matched controls (60 years) (p = 0.961). There were 37 women and 19 men in the control group. The mean number of total postoperative visits per patient was similar between the telehealth (2.6 visits; range: 1-7 visits) and control (2.7 visits; range: 1-6 visits) groups (p = 0.886). The mean time from clinical discharge to as-needed follow-up after surgery was also similar between the telehealth (79.9 days; range: 11-185 days) and control (86.8 days; range: 6-374 days) groups (p = 0.725).

There were two postoperative complications (3.6% of index surgeries) in the telehealth group, namely, one superficial infection and one hypertrophic scar formation. There were four complications (7.1%) in the control group, including two superficial infections, one heterotopic ossification after proximal humerus open-reduction internal fixation, and one carpal tunnel syndrome requiring reoperation after distal radius open-reduction internal fixation. There were no statistically significant differences in complication rates (telehealth = 3.6%, control = 7.1%; p = 0.679) or reoperation rates (telehealth = 0.0%, control = 1.8%; p = 1.00) between the two groups.

## Discussion

Telehealth visits represent resource savings for patients and providers without sacrificing the appropriate quality of care across a myriad of clinical scenarios. Within the postoperative period, the presently identified rate of telehealth visits that required redundant in-person evaluation was less than 2%. The incorporation of telehealth visits did not prolong the postoperative course or add to the average number of postoperative visits performed before clinical discharge. Over half of all patients seen for at least one telehealth visit were discharged to as-needed clinical follow-up directly from their telehealth visit.

In our study, one patient seen via telehealth required redundant in-person evaluation, and the incorporation of telehealth visits did not prolong postoperative courses. The efficacy of postoperative telehealth visits after upper extremity surgery has been established in the literature, and prior investigations support the current findings [4,5]. In a randomized trial comparing telehealth to in-person visits at standardized intervals after rotator cuff repair, Kane et al. found equivalent efficacy between the two groups [4]. Although in-person conversion rates were not directly reported, none of the three reported complications in the telehealth group required in-person evaluation, with only one among the roughly 75 telehealth visits requiring in-person follow-up secondary to technical difficulties [4]. Similarly, Grandizio et al. prospectively assessed 57 patients seen for a single telehealth visit after upper extremity surgery. Four complications were recognized during the virtual encounters and were managed without requiring in-person evaluation [6]. Beyond orthopedic applications, postoperative telehealth visits have been associated with tremendous cost savings [7], and a 2.8% rate of necessary conversion to in-person follow-up [8], similar to the 1.7% rate found in this study. Combining what is now known regarding the efficacy of telehealth visits in the postoperative period, it may be reasonably concluded that telehealth incorporation represents resource savings in the postoperative period after upper extremity surgery. The overwhelming majority of encountered scenarios can be appropriately managed without risking necessary, redundant in-person evaluation.

The comparative benefits and associated patient satisfaction of telehealth visits are also well established in the literature [9-11]. Most significantly, telehealth visits are quicker for both patients and providers and save patients’ travel costs and lost work hours [4-6]. With effective practice implementation, telehealth visits can save substantial resources for the orthopedic practice, including office space, supplies, and ancillary staffing [11,12]. These considerations are crucial within the postoperative global billing period, during which any added healthcare resource utilization will typically not be reimbursed beyond the initially billed surgical bundle payment.

Similarly, it is important to consider the impact that telehealth incorporation may have on patient experience. Patient satisfaction with telehealth is largely comparable to in-person visits [9,13,14], though some investigations have found a higher rate of satisfaction associated with postoperative visits [15]. This is likely attributable, at least in part, to the already established patient-provider rapport compared to new patient visits and the value of decreasing patient burden after surgery. Importantly, the existing evidence suggests that telehealth visits can be incorporated without sacrificing patient satisfaction.

Considering satisfaction, effectiveness, and resource utilization, the postoperative period is likely an ideal application to maximize the benefits associated with telehealth visits. However, to ensure efficacious resource savings, it is critical to tailor therapeutic interventions around or within the virtual visit. In our study, radiographic evaluation was incorporated into 15% of telehealth visits, all scheduled and obtained prior to the visit, and suture removal instructions were given in four visits. Tofte et al. reported the successful incorporation of guided patient suture removal after carpal tunnel release [16]. While such strategies are not universally appropriate for all patients and may be avoided through the use of absorbable sutures in some instances, it is important to consider safe and effective strategies to maximize the seamless addition of telehealth visits within the postoperative course.

There are some limitations to our study beyond its retrospective design. First, there was likely selection bias in which the treating surgeons chose which patients would be seen via telehealth. Second, which postoperative visit (first, second, etc.) was selected by the treating surgeon also introduced a possibility of bias. Third, the included visits took place relatively soon after the widespread adoption of telehealth use, and, as such, the effectiveness of use has likely since improved. Some visits were conducted using audio communication only, which, given the rapid advancements in telehealth platforms, should be avoided. Fourth, complications were rare across both groups, and had they been more common, it is unclear if all would have been recognized during telehealth visits. However, the previously cited literature and complete follow-up through clinical discharge within our study suggest that the risk of this is small. Fifth, the study population included a wide range of surgical procedures. It remains unclear if telehealth visits are more or less effective after certain surgeries. Despite its limitations, this study establishes a rate of redundant in-person evaluation required after postoperative upper extremity telehealth visits and resolves theoretical concerns that telehealth incorporation may prolong postoperative courses or add to the total number of visits required after surgery.

## Conclusions

The rate of necessary in-person follow-up after postoperative telehealth visits was less than 2%. The incorporation of telehealth visits within the postoperative period did not add to the number of visits required after surgery nor did it prolong the postoperative course when compared to matched in-person controls. Concerns that telehealth use may increase healthcare utilization after surgery appear unfounded. Accordingly, the postoperative period is an ideal application for safe and effective telehealth implementation allowing improved optimization of resource allocation.
